# Unpacking Indigenous Social Mobility: Entrepreneurs, Social Networks, and Connections to Culture

**DOI:** 10.1177/00076503231219691

**Published:** 2023-12-28

**Authors:** Rochelle Côté, Michelle Evans

**Affiliations:** 1 Memorial University, St. John’s, Newfoundland, Canada; 2 The University of Melbourne, VIC, Australia; 3 Melbourne Business School, VIC, Australia

**Keywords:** cultural capital, entrepreneurship, indigenous, social capital, social mobility

## Abstract

In settler societies, upward social mobility by Indigenous people is seen in the growth of successful professional and entrepreneurial classes where both wealth creation and social power are significant resources. Yet, public and academic discourses perpetuate the belief that social mobility impacts negatively on Indigenous people by placing cultural identity in conflict with capitalist business practices. Using data from an international comparison consisting of interviews with 220 Indigenous entrepreneurs in research sites across three countries, this article shows that the belief is unfounded and reveals how this duality creates an impossible tension when Indigenous cultural identity is framed as “at risk” because of social mobility. A discursive colonial mind-set remains a central, enduring and problematic organizing principle of the field of Indigenous social mobility, one that requires a shift in the kinds of research questions that are asked and the ways in which social mobility is ultimately defined.

Research and national census data in the United States, Canada, and Australia indicate that Indigenous people in first world nations are increasingly socially mobile. There are a growing number of people with university degrees, levels of income about the national median, employed in professional occupations, and starting businesses (Australian Bureau of Statistics [ABS], 2018; Ruggles et al., 2019; Statistics Canada (StatsCan), 1996-2016). With self-employment positively associated with social mobility and wealth accumulation (Quadrini, 2000), this article focuses on Indigenous entrepreneurship, and Indigenous entrepreneurs, as an entry point to explore the emerging phenomenon of Indigenous social mobility.

Indigenous entrepreneurship has grown at a rate higher than the national averages of these countries (Conway, 2011; StatsCan, 2017b; U.S. Census Bureau, 2019), with a recent Australian study reporting a 4% growth rate per year (Evans et al., 2021). Central to business success is the ability to cultivate social and cultural capital through varied connections and the informal knowledge required to enter those networks and find opportunities (Aldrich et al., 1987; Burt, 1992; Savage, 2015). Complicating this endeavor is persistent inequality of access to networks in a society that continues to be shaped by race (Patfield et al., 2022). Research consistently shows that not all individuals are able to access esteemed networks (Bourdieu, 1986; Coleman, 1988; Tilly, 1998; Wilson, 1987, 1996). Although the impact is well documented for new immigrants and ethnic minorities, we are highlighting that research exploring access to and acquisition of social and cultural capital is limited when it comes to conversations around Indigenous Peoples (Gallagher & Lawrence, 2012; Paradies, 2006; Patfield et al., 2022).

Equally problematic, past theorization argues that there is a disconnect between upward social mobility, and Indigenous social and cultural capital. Walter (2015) provides a nuanced articulation of the distinctiveness of Indigenous social capital, arguing that Indigenous forms of capital are racially stratified against White theoretical conceptions of social and cultural capital, denoting a racial bias. With increased geographical and social mobility, Indigenous people may find themselves occupying urban places, but not inhabiting the same spaces (Walter et al., 2011, p. 9). This is compounded by the pull of Indigenous bonding social capital potentially creating tension with efforts to increase and diversify bridging social capital (Butler & Muir, 2017; Page, 2015). Furthermore, Indigenous experiences of social capital expansion through digital democratization have seen collective Indigenous social networks expand globally through movements like “Social Distance Powwow” and through global collective engagement with Indigenous written and creatively controlled content like Sterlin Harjo’s *Reservation Dogs* (Nungaray, 2022, p. 124). These factors create a relationship between social mobility and social capital that is distinct.

For entrepreneurs, accessing mainstream social and cultural capital resources is thought to increasingly be dependent on integration into the mainstream coupled with distance from relational networks to Indigenous community and culture. Foley and O’Connor (2013, p. 291) argue that Australian Aboriginal entrepreneurs who use bridging ties to the dominant culture to facilitate business necessarily compromise “normative social capital in the indigenous culture.” Even with studies like Gallagher and Lawrence (2012) emphasizing that success in business “did not weaken their perceptions of themselves as Indigenous people” (p. 14), or Gladstone’s (2018) assertion of historical practices of independent trade in Indigenous communities existing alongside collectively owned trading, and digital and popular cultural resistance, debate endures on the negative impact of social mobility to Indigenous cultural identity. Aboriginal academic Larissa Behrendt (2015) reports that with increases in personal income there are decreases in cultural legitimacy. Similarly, for Indigenous entrepreneurs, levels of skepticism and resistance are expressed toward those working on individual-level economic endeavors (Leu et al., 2018). Echoing this in his Narrm Oration in 2016, Wiradjuri journalist Stan Grant posits that the “Indigenous middle class was a phenomenon that is met with suspicion, even hostility” (Grant, 2016; Wahlquist, 2016).

Tensions between collectively held Indigenous values and individual freedom of economic expression is also complicated by cultural norms of Tall Poppy Syndrome, resulting in individuals “cloaking their wealth” to avoid attention and conflict (Warren et al., 2017). Community and collective values often act as a form of regulation on Indigenous entrepreneurial ambition (Colbourne, 2017), especially for enterprises established upon Indigenous value creation activities (Colbourne & Anderson, 2021). Although research shows that generally, there is a positive relationship between middle classness and diverse social and cultural capital, capitalist ideals of success aspired to by Indigenous people are often portrayed as culturally selling out (Bunten, 2011). And further, that success buoyed by increases in mainstream social and cultural capital has a detrimental effect on Indigenous social and cultural capital (Bunten, 2008; Paradies, 2016). In effect, scholars and communities suggest that there are internal and external consequences around acquiring diverse forms of capital and a negative impact of social mobility on Indigenous identities. Here too however, empirical research has yet to look specifically at whether this holds true.

We seek to investigate these areas of inquiry, with the goal of exploring the tension between Indigenous capitalist behavior, exemplified by Indigenous entrepreneurs, and ties to Indigenous networks and culture. This focus will bring original insight on the way that social mobility and economic success are framed in relation to Indigenous people. As such, this article asks: (a) are entrepreneurs, as members of a socially mobile class, cultivating rich and varied social and cultural capital; (b) do mainstream forms of capital come at the expense of Indigenous forms of capital; (c) how is social mobility related to social and cultural capital?

## Emergence of an Indigenous Middle Class

As early as 2000, research reports a growing Indigenous middle class (Côté, 2012; Lahn, 2013; Langton, 2012; Newhouse & Peters, 2003; Satzewich & Wotherspoon, 2000; Wotherspoon, 2003). In part, this middle class is composed of individuals who are educated and successful in professional occupations. Another phenomenon spurring this middle class is the rise of entrepreneurship corresponding with conventional notions of middle class values (Banerjee & Duflo, 2008). Yet traditional sociological conceptions of class and the nature of social and cultural capital that make up our understanding of middle classness are challenged when contextualized by scholars working beyond the west (Neubert, 2022). For example, Global South studies of the phenomenon of Indigenous middle classes emerging from Africa, South America, and Asia are demonstrating the complexity at play between class, race, and identity (Aparicio et al., 2022; Crabtree, 2020; Handley, 2015).

In the United States, Canada, and Australia, Indigenous entrepreneurship is increasingly bound up in open markets of the mainstream economy. Although subsistence trading economies continue to be a vital connection to pre-colonial Indigenous business models, in many developed and democratic nations, access to land and capital to implement primary production business models remains a possibility for only a small percentage of the Indigenous business sector (Evans et al., 2021; Jongwe et al., 2020). Following historical state-based removal and relocation policies across developed and democratic nations including the United States, (see Fixico, 2013), Indigenous entrepreneurs living in cities and engaging in urban economies are incredibly adaptable (Lobo & Peters, 2001). They are creating viable businesses, expanding their social networks, returning to homelands, creating new multi-nation Indigenous communities, inhabiting digital global Indigenous communities, and developing sufficient bridging social capital with urban business people to ensure profitability and longevity in the business world.

As a consequence of both factors that push and pull people to cities, Indigenous people are more representative in urban populations than ever before, with over 65% or more now living in cities across Canada, the United States, and Australia (ABS, 2018; StatsCan, 2017a; U.S. Census Bureau, 2019). Prevailing thinking suggests that because urban Indigenous people are often seen as members of a disadvantaged class (Browne-Yung et al., 2013), individuals experience exclusion from privileged, valued networks in mainstream marketplaces (Evans & Polidano, 2022; Kuhn & Sweetman, 2002). Barriers to mainstream networks are compounded by the legacy and structural nature of racism, alongside the pull of bonding capital developed by Indigenous people and their communities creates “unequal access to economic and cultural capital*”* (Browne-Yung et al., 2013, p. 27).

However, no matter how entrenched the discrimination and inequity, Indigenous resistance and knowledge systems continue to challenge colonial and assimilationist ways of seeing the world. Legal contests have taken the form of de-seating the artifice of *terra nullius* in Australia (Mabo and others v. Queensland, 1992), establishing First Nations sovereignty in Canada (Delgamuukw v. British Columbia, 1997), and establishing the relationship between Indigenous nations, state, and federal government in the United States (Worcester v. Georgia, 1832). This kind of resistance relies on Indigenous people who can be considered empowered within state structures. Individuals who are “educated, employed professionals who often work *with* the state” (Page & Petray, 2015, p. 88), who are also notably members of this emerging middle class. While the nature of poverty and social determinant outcomes associated with Indigenous people demonstrates persistent structural disadvantage experienced by a great percentage of Indigenous people, it is important to consider the experiences of members of all segments of the Indigenous population—including those considered to be socially mobile. How do Indigenous people experience increasing social mobility, and with increased mobility, how do they negotiate connections to mainstream and Indigenous culture and social networks?

## Entrepreneurship and Forms of Capital

On average Indigenous entrepreneurs are first-generation entrepreneurs having grown up in family circumstances that are associated with low social mobility (Frederick & Foley, 2006). Paired with increasing diversity of business interests, educational and professional backgrounds, Indigenous entrepreneurs are increasingly participating in mainstream economies through a range of business ventures (ABS, 2018; StatsCan, 2017b; U.S. Census Bureau, 2019). This participation is further enhanced by the “halo effect” on both services and products produced by Indigenous businesses, a unique source of cultural capital for Indigenous entrepreneurs (Stewart, 2019; Stewart et al., 2014). However, little research has been done to explore the limitations racism plays in the social mobility of racial minorities (Gilbert et al., 2022). Understanding the reproduction and access requirements of privilege and trust through social networks pertaining to wealth, and how that intersects with the composition of Indigenous entrepreneurial social networks is critical for the examination of Indigenous social mobility. Furthermore, entrepreneurship is generally accepted as a vehicle for the upward mobility of individuals and their families (Keister, 2005; Quadrini, 2000), with more recent studies exploring the positive connection between entrepreneurship, income inequality and social mobility (Orozco, 2020; Pathak & Muralidharan, 2017), making Indigenous entrepreneurs an ideal case study.

Although measurements for social mobility continue to privilege Whites and colonial understandings of wealth, and we have argued the limitations of standard conceptions of social mobility, we reason that it is imperative to establish baseline measures of Indigenous social mobility to demonstrate this complex phenomenon before arguing for extending and evolving our understanding of this concept. For this article, we consider three indicators of social mobility—level of education, income, and occupational prestige—and their relationship to diverse social and cultural capital.

Level of education is known to be positively associated with diverse social capital (Erickson, 2004) and business education has been positively associated with entrepreneurship more generally (Do Paço et al., 2015). Indigenous people continue to have lower levels of education (Gray & Beresford, 2008) and there is a negative association between education and participation rates in employment for Indigenous people—low levels of education are linked to low employment rates (Butler & Muir, 2017). Entrepreneurs, however, typically have higher levels of education.

Income has also been found to be positively associated with diverse social capital (Moren Cross & Lin, 2008). As with education, Indigenous people are disproportionately represented in the low socio-economic levels of society, with compounding social determinants being a key consideration (Hajizadeh et al., 2021). When looking to occupational prestige, research shows that people in high prestige occupations are typically better educated, exposed to more diverse experiences, kinds of people, and have higher incomes (Lin, 2001). As a result, their networks are more diverse, increasing the value of their own social capital in turn (Angelusz & Tardos, 2001; Erickson, 2004). Census data show that while Indigenous people continue to be under-represented in high prestige occupations, this is changing, with greater numbers working in professions and also starting businesses. This article examines Indigenous entrepreneurs as a demonstrative socially mobile group by seeking to understand the associations between education, income, occupational prestige, and forms of capital.

Entrepreneurs use the power of bridging social capital resources to access networks, to find opportunities in business and to move between social classes (Cope et al., 2007; Savage, 2015). Bridging, outgroup ties (whether between classes, genders, or racial/ethnic groups) are desirable and essential to move into different classes, promoting values of assimilation as a key to social mobility (Lin, 2001; Putnam, 2000). Research is mixed on whether forming bridging outgroup ties leads to a decline in bonding, ingroup ties (whether within a class, gender, or racial/ethnic group), lending some indirect support to the idea of a disconnect between wealth and Indigenous social and cultural capital (Portes & Zhou, 1993). Research on the African American experience in particular demonstrates that strategic assimilation (Lacy, 2004) demands that great work must be undertaken by individuals and groups as they balance the tightrope between race and class (Benjamin, 1991).

Indigenous entrepreneurs demonstrate highly valued bonded social capital (Levitte, 2004; Light & Dana, 2013) that is enriched via cultural, kinship and family values and norms (Dana et al., 2020), and important personal support (Butler & Muir, 2017). However, for individuals seeking to increase wealth over time, bonding social capital is not sufficient to surmount the challenge of transgressing social strata. Knowing diverse networks of people and institutions matters, with large networks of diverse, high-status contacts reported the best to enhance individual socio-economic advancement (Aldrich et al., 1987; Burt, 1992; Weber, 2009). Knowing people across many different groups allows individuals to effectively move in and out of networks that span across social classes. There are more opportunities, more resources, and in turn, a greater advantage in life when individuals increase their skill at developing their social capital.

Cultural capital has also been shown to be of significant influence on social mobility (Scherger & Savage, 2010). Cultural knowledge and socialization go hand in glove with educational experiences and opportunities alongside parental/familial and teachers’ networks in the development of individual resources. Social and cultural capital also have a mutually reinforcing relationship and are tools that facilitate entry into new groups, and between social classes. Although studies of cultural capital have traditionally considered elite forms of cultural knowledge as the means to boost elite status in society and exclude non-elites from positions of power (Bourdieu, 1986; DiMaggio, 1992), the importance placed on the diversity of the middle class and their tastes is increasing over time (Van Eijck, 1999). Different interests and attributes place people along major lines of stratification like class, gender, and race that shape cultural and social capital, and impact unequally on individual abilities to span social boundaries (Lamont & Lareau, 1988; Portes, 1998; Walter, 2015).

Foley and O’Connor (2013, p. 291) argue that for Indigenous entrepreneurs developing bridging social capital to drive business within dominant cultures compromises social capital within their Indigenous networks. This is further echoed by Walter (2015) who argues that the social capital required to strengthen “new socio-economic positions [leads to] a reduced ability to draw on the support of older social capital assets” such as Indigenous social and cultural connections. For Indigenous people, it is suggested that gaining entrée to predominantly White social groups can come at a cost of being racially and culturally isolated (Walter, 2015, p. 81), or even more distressing “socially sanctioned” for traversing social strata. With a focus on entrepreneurship, we consider whether Indigenous entrepreneurs are truly sacrificing connections to networks and culture for participation in mainstream economies, and whether being social mobile is linked to diverse networks and culture.

## Data and Methods

To study Indigenous entrepreneurship, social and cultural capital, we rely on a mixed method study focusing on factors that contribute to successful business activities in urban marketplaces. The research took place in three large, urban centers in Canada, the United States, and Australia, interviewing respondents about their experiences as Indigenous entrepreneurs, living and working in cities, and who are members of an emerging middle class. With the help of research fellowships to travel and live in these places, in-depth interviews were conducted in Toronto (2008-2009), Phoenix (2011-2012), and Brisbane (2014-2015). It is important to note that achieving a level of equivalency across three nation states proves to be an impossible task; hence the selection of these sites was made based on best contextual match. The contextual criteria considered included cities with diverse economic activity, and diverse populations (ABS, 2017; StatsCan, 2017b; U.S. Census Bureau, 2019); cities with large urban Indigenous populations, and active Indigenous business communities.

This study provides comparable and quantitative measures alongside rich, qualitative data about the experiences of Indigenous entrepreneurs. The data collected includes combined survey and open-ended questions as part of 220 interviews that lasted 1.5 to 4.0 hr with Indigenous entrepreneurs across the three sites. Survey items included indicators of social mobility—education, income—and we were able to code for occupational prestige (please see below for further discussion). The interview guide was replicated across the three sites for consistency, with a structured set of interview questions pertaining to social capital and cultural capital items (the only changes reflecting country-context terminology such as Aboriginal, First Nation, Native American for example), as well as asking Indigenous entrepreneurs about their experiences as business owners.

To ensure as broad a sample as possible, an initial list of entrepreneurs was compiled by using various websites, lists, and searching broadly for Indigenous businesses in each city. Individuals were randomly sampled throughout the compiled list, contacted, and subsequently interviewed. Using a snowball sampling approach, research participants were asked at the end of the interview, for names of anyone else they thought would be good to talk to. This was necessary to adhere to strict timelines for data collection, but to also acknowledge the primacy (and importance) of referral networks within Indigenous communities. In all, the first 30% of respondents were randomly sampled in each site, and the remainder purposefully sample. This led to final response rates between 80% and 90% across all three sites.

Across the three research sites, respondents represent a significantly diverse group (Table 1). Although the majority are members of First Nations, Native American, and Aboriginal nations, they represent between 27 and 48 different nations in Canada, the United States, and Australia, exemplifying the truly diverse nature of urban Indigenous communities. Depending on the city, women entrepreneurs represent just under half of all respondents, with the exception of Brisbane, where they were 63% of the sample. On average, entrepreneurs were around 45 to 46 years of age. Consistent with data around entrepreneurship, respondents’ levels of education were consistently high—with most having some postsecondary. It should be noted here that the distribution around education is not wide. Most entrepreneurs have some form of postsecondary education. When compared with general levels of education for Indigenous people, this is quite different. But as census data show, the number of Indigenous people with university degrees is increasing. We also note that entrepreneurs for this study also report earning around the national average for income. In all, they represent groups of entrepreneurs that are socially mobile but that also vary in terms of the level of social mobility that they experience.

**Table 1.** Demographic Attributes of Sample.

Demographic Attribute	Toronto, Canada	Phoenix, United States	Brisbane, Australia
First Nations, Native American, Aboriginal	92%	98%	80%
Other Indigenous	8% Métis	2% First Nations	15% Torres Strait, 5% other
Indigenous Nations represented	45	27	48
Female	43%	45%	63%
Age (mean)	45 years	46 years	45 years
Level of education (mean)	Some postsecondary	High school	Some postsecondary
Annual income (mean in local $)	CAD$53,000	USD$44,000	AUDS$72,000
Interview years	2008	2011	2014

### Measuring Social Capital

To understand the diversity of an individual’s social networks, our measure of social capital uses an occupational position generator. Originally developed by Lin and Dumin (1986), the position generator asks respondents to indicate whether they know someone that works in each of a list of occupations. Occupational position generators have been used and validated across many contexts and populations, showing that the measure is reliable and consistently measures resource diversity in social networks (e.g., please see Côté, 2023; Erickson, 2004; Miething et al., 2017; Van der Gaag & Snijders, 2005).

To properly understand the diversity of respondents’ social capital and prioritize our interest in knowing whether respondents have rich and varied Indigenous networks, it was necessary to have ethno-racial representation through the position generator. With social boundaries stratifying ethnic groups into occupations, a greater proportion of ethnic minorities and immigrants tend to occupy low-prestige jobs and ethnic economies—contacts known to be less effective in accessing opportunities in mainstream society (Constant & Massey, 2005; Liebig, 2007; Simón et al., 2008). Respondents were asked if they knew someone Indigenous, White, and non-Indigenous/non-White who worked in each of 28 occupations. The number of occupations in which the respondent knew someone in each of these categories was totaled to provide scales for diversity of ties to *Indigenous people*, to *Whites*, and to *non-Indigenous/non-Whites* (proxy for newer immigrant groups). The higher the number of occupations a respondent reports knowing someone, the more diverse the social network and the respondent’s social capital. Occupations vary along a scale of high and low prestige, accounting for differences in status. Prestige scores derived from the cross-national work of Ganzeboom and Treiman (1996) were used to classify occupations along a scale of 0 to 100, or low to high prestige (Table 2).

**Table 2.** Occupational Position Generator (Toronto, Canada).

Do you know someone _______________ in each occupation:
Indigenous, White, Non-Indigenous/Non-White
High prestige occupations^a^
Band Council Member (64)
Hereditary or Elected Chief (71)
Community Economic Development Officer (63)
Venture Capitalist (70)
Lawyer (73)
IT Engineer/Computer Programmer (51)
Sales or Marketing Manager (60)
Entrepreneur/Small Business Owner (50)
Physician (78)
Human Resources Manager (60)
Journalist/Editor in Media (58)
Accountant (62)
Bank Loan Officer (60)
Professor (78)
Government Official (63)
School Teacher (57)
Low-prestige occupations
Policeman/Policewoman (45)
Tailor/Furrier/Dressmaker (40)
Waiter/Waitress (21)
Electrician (44)
Caretaker/Janitor (25)
Hairstylist (32)
Truck Driver (33)
Security Guard (30)
Farmer (40)
Construction Worker (15)
Cashier (34)
Delivery Driver (31)

a Prestige scores in (#).

Occupations also reflect distribution across ethno-racial categories. Care was taken to ensure that the high and low-prestige occupations included some with a higher percentage of workers who are Indigenous, and who are members of other ethnic minorities. In addition, three important positions in Indigenous communities were also included (Hereditary or Elected Chief, Band Council Member, Indigenous Community Economic Development Officer), even though they do not fit within standard occupational categories. In certain cases, occupations with similar prestige scores were substituted to reflect a specific country context. An example would be delivery driver (Canada), casino worker (United States), and mine worker (Australia).

### Measuring Cultural Capital

The cultural capital generator uses a list of cultural activities and events and asks respondents if they have participated in activities over the past year (Table 3). A range of both high and low-prestige activities were included as generally ranked in the literature (DiMaggio, 1992). It also incorporated activities that could be considered exclusively Indigenous. In total, respondents were asked to indicate whether they participated in 19 Indigenous activities and 15 non-Indigenous, with columns for White/mainstream, and for non-Indigenous/non-White activities. The number of activities in which the respondent participated was totaled to provide scales for diversity of Indigenous, Mainstream, and Other Ethnic (non-Indigenous/non-White) cultural capital. The higher the reported number of activities participated in, the more diverse the respondent’s cultural capital.

**Table 3.** Cultural Capital Generator (Brisbane, Australia) Have you engaged in the following activities during the past year? Please CHECK in the appropriate column if you have participated or engaged in an activity that is:

Activity	A. Indigenous	B. White/ “Mainstream”	C. Non-Indigenous/Non-White
A. NAIDOC Week	1□	Not applicable	
B. Seen a movie in a movie theater	1□	2□	3□
C. Gone to a live music performance	1□	2□	3□
D. Attended a live performance of a non-musical stage play	1□	2□	3□
E. Watched a live ballet or dance performance	1□	2□	3□
F. Visited an art museum or gallery	1□	2□	3□
G. Read a novel, poem or play by a ___________ author	1□	2□	3□
H. Eaten out at a restaurant serving _____________ foods	1□	2□	3□
I. Shopped in a store selling ___________ goods	1□	2□	3□
J. Taken part in community or political activities? i.e.: elections, fundraiser	1□	2□	3□
K. Taken part in Indigenous cultural/traditional activities?	1□	Not applicable	
L. Taken part in mainstream or other ethnic cultural activities? i.e.: Australia Day, Chinese New Year’s, Anzac Day, Diwali, etc.	Not applicable	2□	3□
M. Heard a classical music or opera performance	1□	2□	3□
N. Watched a television show	1□	2□	3□
O. Listened to a radio program	1□	2□	3□
P. Attended a sporting event	1□	2□	3□
Q. Purchased art	1□	2□	3□
R. Read a magazine	1□	2□	3□
S. Attended IBCA National Indigenous Business Association Conference	1□	Not applicable	
T. Attended the Laura Aboriginal Dance and Cultural Festival	1□		
U. Attended the Torres Strait Cultural Festival	1□		
V. Attended the Cairns Indigenous Art Fair	1□		
W. Participated in the collection, preparation, and eating of bush tucker	1□		

Income was included as an ordinal variable with 10 income categories: Less than US$20k = 1; US$20k-US$29,999 = 2; US$30k-US$39,999 = 3; US$40k-US$49,999 = 4; US$50k-US$59,999 = 5; US$60k = US$69,999 = 6; US$70k-US$79,999 = 7; US$80k- US$89,999 = 8; US$90k-US$99,999 = 9; US$100k or more=10. For analysis, income was split at the 50th percentile. Level of Education is an ordinal variable with six levels: less than high school = 0, high school = 1, some technical school or university =2, completed technical = 3, completed bachelor = 4 and completed post graduate = 5. A series of dummy variables were created for the regression analysis, with “high school or less” as the left-out category.

Although it is the case that all respondents are entrepreneurs and business owners, they also have occupations as business owners, and/or represent occupations for the business. As such, we were able to code for the prestige of the occupation (Occupational Prestige) that each respondent held, in addition to being a business owner. This allowed us to look at another indicator of social mobility, by considering the prestige of respondent occupations and its association with the diversity of social capital. Using Ganzeboom and Treiman (1996), cross-national prestige scores were derived for each, based on the occupation that the respondent held or that the business represented. Prestige scores range from 0 to 100 and were integrated into the regression as a continuous variable.

### Control Variables—Respondent and Business Attributes

City of interview was included in the ordinary least squares (OLS) regression tables to control for any differences that may have occurred due to location. A series of dummy variables were created, where Toronto, Canada was the left-out category. Gender is as a dummy variable coded 1 for “Female.” Age in years was included as a continuous variable. Having a marital or common-law partner (Partner) may also factor in increasing diversity of social and cultural capital by being an added source of network diversity. They may have access to ties with resources and new opportunities that the entrepreneur can tap into. Participation in voluntary associations was also included as a measure of how many associations individuals were active in over the past 5 years. Voluntary association participation was included as a dummy variable, where high participation was coded as 1. High participation was determined by locating the mean, which for the combined sample was equaled to participation in two voluntary associations or more.

We also control for a range of business characteristics. Using the North American Industry Classification system (NAICS), businesses were initially classified by industry as participating in primary (mining, forestry, agriculture), secondary (manufacturing or processing) or tertiary (sales or service) industries (U.S. Census Bureau, 1997). NAICS is a measure that produces a standardized code for similar occupations across the United States and Canada, allowing for comparison across the two nations. The Australian and New Zealand Standard Industrial Classification purposefully aligns with NAICS categories, to explicitly allow for international comparisons (ABS, 2013). As such, we confidently coded business into one of three industry categories. Primary and secondary industry participants were collapsed into one category given the number of entrepreneurs whose business fit, and then used as the left-out category in the regression analyses.

Formal support was measured as the total number of business assistance programs entrepreneurs used throughout the course of their business. Fifteen government, non-profit, and private sources were identified and listed, corresponding with the country context. These ranged from banks, government programs and agencies, online business networks, chambers of commerce, and business associations.

It was also important to consider market reach as a percentage of business that entrepreneurs were doing in a variety of markets. Arguably, business success/impact is tied to geographical range of sales—the larger the range, the greater the impact. As part of the survey, respondents were asked what percentage of their sales came from (a) Cities: Toronto, Phoenix, or Brisbane Metropolitan Areas (b) province, state, native nations (c) nationally (d) internationally. Combined, these four categories totaled 100% for each respondent. Sales within the province/state are considered to have greater impact than within cities, national better than province/state and cities, and international better than national, province/state, and city.

## Data Analysis Strategies

Two statistical procedures were used to analyze the cross-site survey data. We first used paired-sample *t*-tests to compare for significant differences between means for diversity of social capital. We compared (a) mean diversity of ties to Indigenous people and to Whites, (b) mean diversity of ties to non-Whites and to Whites, (c) mean diversity of ties to Indigenous people and to non-Whites, and (d) mean diversity of forms of social capital between the three research sites. We then used paired sample *t*-tests to test for significant differences between means for diversity of cultural capital. We compare the diversity of cultural capital of three ethno-racial groups, (a) mean diversity of Indigenous to mainstream cultural capital, (b) mean diversity of other ethnic to mainstream cultural capital, (c) mean diversity of Indigenous to other ethnic cultural capital, and (d) mean diversity of forms of cultural capital between the three research sites.

OLS regressions were then used to consider the association between indicators of social mobility, and diverse social and cultural capital, controlling for respondent and business attributes. To allow for comparison across ethno-racial forms of capitals, we report unstandardized coefficients (*B)*, with standard errors (SE) reported underneath.

The analysis of the qualitative data started with the recording of responses in the Canadian interviews. A separate file was created for each question in the interview guide, where transcribed responses were cut and pasted into the file. With the documentation of responses from each question, active reading and an inductive approach was used to identify themes in the data. Of note, open-ended interview questions were crafted in order to elicit the greatest range of responses possible. Within each question document, themes were tracked in terms of the frequency of relevant keywords, phrases, ideas emerging across respondents. These themes were then used as the basis for a conceptual content analysis of both the American and Australian interviews, while also attending to new themes in these datasets. Of particular importance to this current article, were themes that consistently emerged around how Indigenous entrepreneurs define and experience success.

The general focus of this study was looking at how Indigenous entrepreneurs engaged with large, urban marketplaces. Within the context of the overall project, respondents were asked how they defined business success, and/or what needed to happen in order for them to consider their businesses a success, as part of their experiences as Indigenous entrepreneurs. While entrepreneurial success is characteristically defined in socio-economic terms, five general themes emerged through interviews that differ in some ways from traditional thinking. Indigenous respondents link success to (a) Responsibility to community, (b) Responsibility to family and friends, (c) Ability to pay bills, (d) Ability to do what you want and be happy, (e) Business does well, make lots of money, and have recognition. Several sub-themes were uncovered that are attached to each of these main themes (see Table 8). Although this article does not deal specifically with Indigenous conceptions of business success, the first two themes around how respondents define success provide context, and speak directly to the primacy of entrepreneurs’ ties to family, friends, and culture, or their social and cultural capital. It is for this reason, that we introduce and discuss the first two themes around success here. The much larger topic of Indigenous definitions of success remains the topic of another paper.

## Findings

The primary function of social capital and cultural capital, and its relationship to upward social mobility, is establishing and replicating connections to social, cultural, and economic structures in society (Bourdieu, 1986). Little research exists to examine Indigenous experiences with social mobility, social and cultural capital. The findings of our study go some way to demonstrating the rich and varied social and cultural capital of entrepreneurs as representatives of the Indigenous socially mobile.

Although we cannot look directly at the causal effect of increasing social mobility on forms of capital without the benefit of longitudinal data, we present data that (a) compares the diversity of entrepreneurs’ European/mainstream social and cultural capital with the relative diversity of Indigenous social and cultural capital, and that (b) measures indicators of social mobility and their relationship to social and cultural capital diversity. We also present findings from the qualitative portion of the interviews that speak to the importance entrepreneurs place on maintaining and developing their connections to Indigenous family, friends, communities, and culture as part of their success as business owners—an indicator that Indigenous social and cultural capital are top of mind.

### Are Entrepreneurs Cultivating Rich and Varied Social and Cultural Capital?

Contrary to prevailing theory, we find that there is neither a negative or significant relationship between mainstream and Indigenous social and cultural capital. Using paired sample t-tests, we find that there is no significant difference between mean diversity of ties to Indigenous people and mean diversity of ties to Whites (t_215_=1.38; *p*<.17). Indigenous entrepreneurs are connected to a diverse range of both Indigenous people and Whites (individuals of European descent), knowing people in 11 to 12 different occupations, respectively (Table 4). Interestingly, mean diversity of ties to non-Indigenous/non-Whites are significantly less diverse than ties to Whites, and to Indigenous people (t_216_= 21.44; *p*<.001; t_215_= 21.26; *p*<.001). Indigenous respondents only know, on average, non-Indigenous/non-Whites in three occupations. This general pattern shows that Indigenous entrepreneurs across the three research sites are well connected to diverse Indigenous networks, while cultivating connections within non-Indigenous networks to significantly different degrees.

**Table 4.** Paired Sample t-Test Results for Mean Diversity of Social Capital.

	*M*	*SD*	SE mean	Paired *t*-test		
				*t*-value	*df*	Sig (two-tailed)
Pair 1						
*Diversity of Ties to Indigenous People	11.69	6.07	0.41	1.38	215	0.17
Diversity of Ties to Whites	10.89	6.37	0.43			
Pair 2						
Diversity of Ties to Whites	10.89	6.37	0.43	21.44	216	0.00
Diversity of Ties to Non-Whites	2.78	3.76	0.26			
Pair 3						
Diversity of Ties to Indigenous People	11.69	6.07	0.41	21.26	215	0.00
Diversity of Ties to Non-Whites	2.78	3.76	0.26			

* Diversity = total # known in a list of 28 occupations.

Looking across the three research sites, there are some differences in the diversity of social capital across Indigenous entrepreneurs (Table 5). Paired sample *t*-tests show that when it comes to diversity of ties to Indigenous people, there are significant differences between respondents in Toronto and Brisbane (t_152_=2.69; *p*<.008), and Phoenix and Brisbane (t_134_=3.07; *p*<.003). Entrepreneurs in Phoenix and Toronto know a greater number of Indigenous people across occupations, than do entrepreneurs in Brisbane.

**Table 5.** Paired Sample t-Test Results for Mean Diversity of Social Capital Across Cities.

	Diversity of ties to Indigenous people				Diversity of ties to whites				Diversity of ties to non-whites			
	*M* (*SD*)	*t* value	*df*	Sig (two-tailed)	*M* (*SD*)	*t* value	*df*	Sig (two-tailed)	*M* (*SD*)	*t* value	*df*	Sig (two-tailed)
**Pair 1**												
Toronto	12.29 (*6.06*)	−0.678	140	.499	11.62 (*6.38*)	2.617**	140	.010	2.83 (*4.03*)	0.486	140	.628
Phoenix	13.03 (*7.02*)				8.76 (*6.53*)				2.50 (*3.86*)			
**Pair 2**												
Phoenix	13.03 (*7.02*)	3.069**	134	.003	8.76 (*6.53*)	−2.935**	134	.002	2.50 (*3.86*)	−0.763	134	.447
Brisbane	9.92 (*4.75*)				11.87 (*5.86*)				2.97 (*3.39*)			
**Pair 3**												
Toronto	12.29 (*6.06*)	2.69**	152	.008	11.62 (*6.38*)	−0.258	152	.797	2.83 (*4.03*)	0.247	152	.805
Brisbane	9.92 (*4.75*)				11.87 (*5.86*)				2.97 (*3.39*)			

* *p* < .05. **p < .01. ***p < .001.

Looking at ties to Whites, findings show that respondents in Toronto and Brisbane have significantly more diverse social capital than respondents in Phoenix (t_140_=2.62; *p*<.01; t_134_= −2.94; *p*<.002). Entrepreneurs in Toronto and Brisbane report knowing individuals of European descent across a wider range of occupations, than entrepreneurs in Phoenix. When looking at ties to Non-Whites across the three research sites, there are no significant differences. Irrespective of where entrepreneurs are located, ties to Non-Whites are similarly diverse, with entrepreneurs knowing individuals across only three occupations.

A similar story emerges when considering Indigenous entrepreneurs’ cultural capital, or participation in cultural activities (Table 6). Paired sample *t*-tests reveal significant differences between mean diversity of Indigenous and mainstream cultural capital (t_211_=3.64; *p*<.001). Indigenous entrepreneurs participate in a significantly greater range of Indigenous cultural activities, when compared with mainstream cultural activities. When considering participation in cultural activities linked to newer immigrant groups however, participation is much lower. In fact, respondents only participate in an average of four “Other Ethnic” cultural activities compared with mainstream and Indigenous cultural activities (t_211_=14.45; *p*<.001; t_212_=20.70; *p*<.001).

**Table 6.** Paired Sample t-Test Results for Mean Diversity of Cultural Capital.

				Paired *t*-test		
	*M*	*SD*	SE mean	*t* value	*df*	Sig (two-tailed)
Pair 1						
*Diversity of Indigenous cultural capital	9.40	4.24	0.29	3.64	211	.00
Diversity of mainstream cultural capital	8.18	3.34	0.23			
Pair 2						
Diversity of mainstream cultural capital	8.18	3.34	0.23	14.45	211	.00
Diversity of ethnic cultural capital	3.85	3.61	0.25			
Pair 3						
Diversity of Indigenous cultural capital	9.40	4.24	0.29	20.70	212	.00
Diversity of ethnic cultural capital	3.85	3.61	0.25			

* Diversity = total # participated in a list of cultural activities.

Looking across the research sites, we also find differences in mean levels of cultural capital (Table 7). Looking between respondents in Toronto and Phoenix, we find that entrepreneurs in Toronto are participating in a significantly greater range of Indigenous and “other ethnic” cultural activities, compared with entrepreneurs in Phoenix ((t_137_=2.52; *p*<.007; t_137_=2.64; *p*<.009). Conversely, respondents in Phoenix have significantly more diverse mainstream cultural capital, than do respondents in Toronto, participating in a greater range of mainstream cultural activities ((t_137_=−9.94; *p*<.001).

**Table 7.** Paired Sample t-Test Results for Mean Diversity of Cultural Capital Across Cities.

	Diversity of indigenous cultural capital				Diversity of mainstream cultural capital				Diversity of ethnic cultural capital			
	*M* (*SD*)	*t* value	*df*	Sig (two-tailed)	*M* (*SD*)	*t* value	*df*	Sig (two-tailed)	*M* (*SD*)	*t* value	*df*	Sig (two-tailed)
**Pair 1**												
Toronto	10.32 (4.33)	2.516**	137	.007	5.71 (2.23)	−9.942***	137	<.001	4.49 (3.70)	2.637**	137	.009
Phoenix	8.52 (3.96)				10.24 (3.12)				2.85 (3.56)			
**Pair 2**												
Phoenix	8.52 (3.96)	−0.771	132	.442	10.24 (3.12)	2.041*	132	.043	2.85 (3.56)	−1.950	132	.053
Brisbane	9.07 (4.23)				9.18 ( 2.86)				4.03 (3.40)			
**Pair 3**												
Toronto	10.32 (4.33)	1.803*	151	.037	5.71 (2.23)	−8.382***	151	<.001	4.49 (3.70)	0.810	151	.419
Brisbane	9.07 (4.23)				9.18 ( 2.86)				4.03 (3.40)			

* *p* < .05. **p < .01. ***p < .001.

Similarly, when we compare Toronto and Brisbane, we find differences in the mean level of Indigenous and mainstream cultural capital. Respondents in Toronto are participating in a greater range of Indigenous cultural activities than respondents in Brisbane (t_151_=1.80; *p*<.04). Conversely, entrepreneurs in Brisbane have significantly more diverse mainstream cultural capital than entrepreneurs in Toronto, participating in a greater range of mainstream activities (t_151_=−8.38; *p*<.001).

Looking to mean diversity of cultural capital between entrepreneurs in Phoenix and Brisbane, we find that there are no significant differences for Indigenous and ethnic cultural capital. For mainstream cultural capital, respondents in Phoenix have significantly more diverse cultural capital than respondents from Brisbane, participating in a greater range of mainstream activities (t_132_=2.04; *p*<.04).

In general, we find that Indigenous entrepreneurs have diverse Indigenous and White (European) social capital, and Indigenous and mainstream cultural capital. Although *t*-tests show that the difference is significant for these forms of cultural capital, this is not the case for social capital. Differences also exist across research sites. Entrepreneurs in Toronto, Phoenix, and Brisbane do have variable levels of social and cultural capital, when compared with each other.

What does this all mean when it comes to White/mainstream social/cultural capital replacing Indigenous social/cultural capital, as a product of being socially mobile and assimilating? Without longitudinal data that tracks Indigenous entrepreneurs over a significant period, we cannot discuss cause, but we can look at the importance that respondents place on maintaining Indigenous social and cultural capital as a proxy.

### Do Mainstream Forms of Capital Come at The Expense of Indigenous Forms of Capital?

When asked in interviews, how and when they would consider themselves to be successful as entrepreneurs, respondents were very clear about the importance of maintaining connections to their Indigenous communities, providing for family and friends, and giving back to their Indigenous communities as a marker of success. In this respect, their Indigenous social and cultural capital were top of mind, and their success as business owners the vehicle for realizing the strengthening of these connections (Table 8, Theme 1, and Theme 2). This was true of entrepreneurs in Toronto, Phoenix, and Brisbane.

**Table 8.** Themes on the Relationship Between Social Mobility and Forms of Capital.

Theme	Sub-themes	“Success is . . .” (City, Participant number)
1. Responsibility to community	a. Providing jobs to community	“. . . getting about 30% Indigenous employment. That’s what we’re all about. I just want to get black fellas working in our business.” (Brisbane, 21)
	b. Act as mentor	“. . . if I can get at least one or two employees out of five or six that are Native American employees . . . and these employees . . . can go on their own to do their own stuff. I think it all starts at the . . . college level or the high school level. To be able to be there to mentor . . .” (Phoenix, 16)
	c. Providing a future for Indigenous Peoples/communities	“. . . helping with some of the big issues like housing, education, and dollars for native communities. Success is really built around getting tangible things for people.” (Toronto, 22)
	d. Change public perception of Indigenous Peoples	“. . . changing the attitudes and that’s what I get for us, I get the hope that comes in. We’re changing the attitudes so I’m on the right track . . . That discrimination will always be there with whoever you deal with, but once they see who you are and meet you and start talking to you their attitudes start to change and they want to help. As an Aboriginal person, changing attitudes is number one.” (Brisbane, 58)
2. Responsibility to family and friends	a. Providing a home	“. . . being able to pay for my parent’s house . . . I don’t mind giving back whether it’s to my family or others who sort of helped.” (Brisbane, 12)
	b. Providing a role model	“. . . really for my son to see what success is for his mommy.” (Toronto, 4)
	c. Ability to take vacations, have the extras that other families can have	“. . . supply[ing] my family with everything they need. Not everything they want, but everything they need and a little want, then I’m a happy man.” (Brisbane, 6)
3. Ability to pay bills	a. Financial stability and security	“. . . for the business to make enough money to pay my bills, to make enough money to provide some security for my family.” (Toronto, 44)
	b. Get off welfare, the dole	“. . . surviving, my five-year plan is to get out of debt. It’s been going on for more than five year it’s been eleven years now I’ve been trying to get out of debt.” (Toronto, 25)
	c. Not about making lots of money, or being a millionaire	“. . . my confidence level that I’ve had. Happiness doesn’t rely on my bank account.” (Brisbane, 28)
4. Do what you want to do and being happy with it, work hard	a. Having work that has meaning	“. . . living with no regrets and doing what I love. Somehow or other what I’m doing is making the world a little bit better.” (Phoenix, 5)
	b. Feeling fulfilled at the end of the day	“. . . knowledge. Knowledge and I think being able to say I am happy doing what I’m doing. That’s all.” (Phoenix, 7)
	c. Plan own hours, schedule, flexibility	“. . . not having to work like a dickens because life’s short, and having some choice over that. I guess another word for it is freedom. Extra freedom.” (Brisbane, 40)
5. Business does well, growing, make money, recognition, longevity	a. Financial wealth	“. . . making a lot of money so then I can step away and leave some really good managers to manage it, and I still oversee it. I can do whatever I want.” (Brisbane, 2)
	b. Recognition as leader, good at what you do	“. . . recognition from other writers, from readers. I guess that’s one reason I keep entering those silly awards is because that gives me an opportunity to have my work recognized.” (Phoenix, 51)
	c. Seen as on par with others who are also considered to be leaders in their industry	“. . . hav[ing] a good name for yourself that people know who you are, have a reputation you know what I mean, a good reputation. Yeah, I think if somebody knows who you are then you can be successful.” (Brisbane, 36)

Social mobility is seen as a vehicle for self-determining, and success is defined over and over in the interviews as being responsible to family, friends, and to their Indigenous communities. This took the form of providing jobs, mentoring future generations, and changing the public perception of Indigenous Peoples. “We are still helping with some of the big issues like housing, education, and dollars for native communities. Success is really built around getting tangible things for people,” says Luke. Dennis agrees, “. . . Another success [for me] is getting about 30% Indigenous employment. That’s what we’re all about. I just want to get black fellas working in our business.”

Michael states, “My success would be to see my children and my grandchildren still hold the culture and so my wife and I could just travel around and enjoy the rest of our lives together and help people when they need it. That’s success, that’s support of people.” Indigenous entrepreneurs are embedded in their Indigenous communities through their family and kinship networks, practicing community and cultural values, and strengthening their bonding social capital.

Entrepreneurs also stressed the importance of mentoring future generations, “The truth of my mandate is the mentorship and the helping and all those sorts of things in our community, but the business is the enabler for me, long term,” says Brendan, or Stanford who states, “If I can get more into this profession then at least I’m successful . . . I’d say if I can get at least one or two employees out of five or six that are Native American employees. I think it all starts at the . . . college level or the high school level. To be able to be there to mentor.” Paul B. exemplifies this attitude, “[I] try to give people a hope when I go home. I don’t lecture them, I don’t judge them, I just try to say I’m doing OK . . . and, how can I help? It’s peer support back to my friends I grew up with who weren’t as lucky as me.” Mentoring is a way of connecting established or emerging entrepreneurs, or younger people who have an interest in the business or profession. It is a way of creating social networks and developing social capital between generations and passing on knowledge or cultural capital.

Culture provides grounding for entrepreneurs, where they also see a responsibility to their Indigenous community to educate the public and change perceptions of Indigenous people with culture and personal connection. Says Millie,

I want to . . .change [the] public perception of native people [and] get beyond the stereotypes. I think some of the public are not aware . . . that native people still exist so it gets as basic as that. If I can say we still exist. I had a . . . friend who when he met me said, “I thought you people were extinct.” We’re not extinct I am standing right here! [The] National Museum of the American Indian published a book called, “Do Natives Still Live in Tepees?” That question is asked by school children to this day, do native people live in tepees. So success to me would be to change that perception of native people.

Karen agrees,

It’s changing the attitudes and that’s what I get for us, I get the hope that comes in. We’re changing the attitudes so I’m on the right track . . . That discrimination will always be there with whoever you deal with, but once they see who you are and meet you and start talking to you their attitudes start to change and they want to help. As an Aboriginal person, changing attitudes is number one.

Joseph also states, “All I want to do is change [the] public perception of native people [to] get beyond the stereotypes.” Far from being kept out of the way when doing business, Indigenous social and cultural capital is front and center for entrepreneurs as they take on the responsibility of their communities to change public perceptions.

In a personal way, entrepreneurs also see a responsibility specifically to their families and friends. Craig states, “I supply my family with everything they need. Not everything they want, but everything they need and a little want, then I’m a happy man, that’s it.” Neil says, “I love the fact that I was able to pay for my parent’s house . . . I don’t mind giving back whether it’s to my family or others who sort of helped.” And finally, Jeremy says, “being able to support my family, being able to support my extended family . . . providing opportunities for education for my nephews and nieces that weren’t necessarily there. Being able to give my parents and in-laws a place to live. A safe home . . .”

What becomes evident is the importance that respondents place on maintaining their Indigenous social and cultural capital, but also in using their social mobility to help their communities, families, and friends. In this respect, sustaining and strengthening connections to their Indigenous communities, and using their social mobility to help, was fundamental to how entrepreneurs defined success. Diverse social networks and cultural participation are important to achieving these goals, and respondents were clear that they are actively maintaining and building these—Indigenous social and cultural capital is front of mind.

Our findings suggest that Indigenous entrepreneurs have diverse connections to Indigenous networks and culture, as well as diverse non-Indigenous networks and culture. But how is social mobility related to social and cultural capital? While general research around social mobility and forms of capital suggest a positive relationship, theories and claims around Indigenous social mobility suggest a negative one—the diversity of Indigenous social and cultural capital should decrease with increases in levels of education, occupational prestige, and income (Behrendt, 2015; Bunten, 2011; Foley & O’Connor, 2013; Gladstone, 2018; Paradies, 2016; Walter, 2015; Walter et al., 2011). The assumption is that social mobility comes at the expense of assimilating away from Indigenous social networks and culture.

### How Is Social Mobility Related to Social and Cultural Capital?

Using OLS regression, we can test theories around the incompatibility of social mobility and maintaining Indigenous social networks and cultural practices. We control for business and personal attributes, and include a series of dummy variables to account for the relative effect of research site, to test the relationship of social mobility proxies—occupational prestige, income, education—to determine whether an increase in these factors is associated with a similar decline in Indigenous social and cultural capital, and increase in mainstream social and cultural capital.

Focusing first on social capital, we find that with increasing levels of occupational prestige and levels of income, there are also significant increases in the diversity of Indigenous social capital (Table 9). Indigenous entrepreneurs with higher levels of income also have increasingly diverse Indigenous social capital, knowing people across a greater number of occupations. Similarly, respondents with occupations associated with higher levels of prestige also have more diverse Indigenous social capital. Education is negatively but not significantly associated with Indigenous social capital, suggesting that irrespective of the level of education, respondents have similarly diverse ties to Indigenous people.

**Table 9.** OLS Regression of Factors Associated With Diverse Social Capital.

	Diverse ties to Indigenous people	Diverse ties to whites	Diverse ties to non-whites
**Independent variables**	*B(s.e.)*	*B(s.e.)*	*B(s.e.)*
Level of education (high school or less)			
Some postsecondary	−0.221 (1.50)	−0.259 (1.53)	0.892 (1.01)
Postsecondary	−0.420 (1.44)	−0.056 (1.47)	0.636 (0.97)
Postgraduate	−2.122 (1.70)	−1.121 (1.73)	−0.290 (1.14)
Occupational prestige	0.103* (0.40)	0.011 (0.04)	0.029 (0.03)
Income above 50th percentile	1.919* (0.93)	2.034* (0.94)	0.485 (0.62)
**Controls—business attributes**			
City (Toronto, ON)			
Phoenix, USA	1.258 (1.15)	−3.513** (1.17)	−0.572 (0.77)
Brisbane, QLD	−2.399* (1.04)	−0.690 (1.06)	−0.180 (0.70)
Industry (primary/secondary)			
Tertiary, goods and services	0.333 (1.05)	−0.610 (1.07)	−0.920 (0.70)
Formal support, business assistance	0.046 (0.28)	−0.609* (0.29)	−0.237 (0.79)
Main market (province/state)			
National, international	2.057* (0.93)	1.077 (0.95)	0.946 (0.63)
City	−1.081 (1.21)	2.088 (1.23)	0.801 (0.81)
**Controls—respondent attributes**			
Female	−0.561 (0.90)	0.767 (0.92)	0.406 (0.61)
Age	−0.018 (0.05)	0.023 (0.05)	0.003 (0.03)
Partner	−1.081 (1.00)	2.664** (1.02)	0.659 (0.68)
Voluntary association participation ≥2	−0.361 (0.90)	1.582 (0.92)	0.325 (0.61)
**(Constant)**	6.658**	5.539***	−0.654
**Adjusted** *R* ^2^	.084	.013	−.007
*N*	191	191	191

* *p* < .05. **p < .01. ***p < .001. OLS = ordinary least squares.

Looking at the effect of research site on the diversity of Indigenous social capital, entrepreneurs in Brisbane have significantly less diverse Indigenous social capital, when compared with entrepreneurs in Toronto. Respondents living and working in Brisbane know fewer Indigenous people across occupations. The diversity of Indigenous social capital is also significant related to the marketplaces they do business in. Respondents who increasingly do business nationally or internationally, also have significantly more diverse ties to Indigenous people, compared with entrepreneurs who do more business within provincial or state borders.

When we look to diverse ties to Whites, we find that level of income is positively and significantly related. Respondents with higher levels of income, also have more diverse ties to Whites, or individuals of European ancestry. Occupational prestige, while positively related to White social capital, is not significant. Respondents with occupations across the scale of prestige have equally diverse social capital. We find a similar relationship with differing levels of education.

We do find a difference when it comes to looking across the research sites. Indigenous entrepreneurs in Phoenix have significantly less diverse ties to Whites than entrepreneurs in Toronto. We also find a negative effect of formal support—entrepreneurs who increasingly avail of business assistance programs have less diverse ties to Whites. Finally, respondents who are married or have a domestic partner, also have significantly more diverse ties to Whites, than individuals who do not.

When it comes to developing increasingly diverse non-Indigenous/non-White social capital, findings show a mostly positive relationship with increasing social mobility. The relationships are not significant however, suggesting that irrespective of increases in education, income or occupational prestige, respondents have similarly diverse ties to new immigrant groups. Combined with evidence from the paired sample t-tests, findings from our OLS regression show that there is no significant effect of research site, when it comes to diverse non-White/non-Indigenous social capital.

We find somewhat similar results when we look at how indicators of social mobility are related to cultural capital (Table 10). Irrespective of whether we look at level of education, occupational prestige, or level of income, respondents have similarly diverse cultural capital. One exception, we find that individuals with occupations considered to have greater levels of prestige, also have significantly more diverse Indigenous cultural capital. In other words, they participate in a greater range of Indigenous cultural activities than respondents with occupations considered to have lower prestige.

**Table 10.** OLS Regression of Factors Associated With Diverse Cultural Capital.

	Diverse indigenous cultural capital	Diverse mainstream cultural capital	Diverse ethnic cultural capital
	*B (SE)*	*B (SE)*	*B (SE)*
**Independent variables**			
Level of education (high school or less)			
Some postsecondary	1.480 *(1.04)*	0.257 *(0.68)*	1.087 *(0.92)*
Postsecondary	0.389 *(0.99)*	0.795 *(0.65)*	0.786 *(0.88)*
Postgraduate	1.014 *(1.17)*	0.601 *(0.76)*	0.793 *(1.04)*
Occupational prestige	0.091*** *(0.03)*	0.020 *(0.02)*	0.029 *(0.02)*
Income above 50th percentile	0.783 *(0.63)*	0.796 *(0.41)*	0.390 *(0.56)*
**Controls—business attributes**			
City (Toronto, ON)			
Phoenix, USA	−1.041 *(0.79)*	5.071*** *(0.52)*	−1.580* *(0.71)*
Brisbane, QLD	−0.813 *(0.71)*	3.649*** *(0.46)*	−0.087 *(0.63)*
Industry (primary/secondary)			
Tertiary, goods and services	−0.327 *(0.72)*	0.285 *(0.47)*	−0.217 *(0.64)*
Formal support, business assistance	0.276 *(0.19)*	−0.067 *(0.13)*	0.168 *(0.17)*
Main market (province/state)			
National, international	1.716** *(0.64)*	1.098** *(0.42)*	0.559 *(0.57)*
City	0.345 *(0.82)*	0.486 *(0.53)*	1.092 *(0.73)*
**Controls—respondent attributes**			
Female	−0.629 *(0.61)*	0.162 *(0.40)*	0.027 *(0.55)*
Age	−0.047 *(0.03)*	−0.043* *(0.02)*	−0.016 *(0.03)*
Partner	−0.523 *(0.69)*	−0.177 *(0.45)*	−1.044 *(0.61)*
Voluntary association participation ≥2	1.214* *(0.62)*	0.612 *(0.40)*	−0.043 *(0.55)*
**(Constant)**	3.830***	3.780***	1.593
**Adjusted** *R* ^2^	.147	.413	.042
*N*	188	188	188

* *p* < .05. **p < .01. ***p < .001. OLS = ordinary least squares.

Reflecting the findings of paired sample *t*-tests in Table 7, living and working in Phoenix and Brisbane are both significantly related to a greater diversity of mainstream cultural capital, when compared with living and working in Toronto. Respondents in Phoenix and Brisbane participate in a greater number of mainstream cultural activities, compared with respondents in Toronto. Conversely, we find a significant and negative effect of Phoenix, when it comes to diverse ethnic cultural capital, compared with Toronto. Indigenous entrepreneurs living and working in Toronto participate in a greater range of cultural activities associated with newer immigrant groups.

We again find a positive and significant effect of national and international market activity. When compared with entrepreneurs who have a higher percentage of business within provincial and state boundaries, respondents who increasingly do business on a larger scale also participate in an increasingly diverse range of Indigenous and mainstream cultural activities. As entrepreneurs age, findings show a negative but significant association with mainstream cultural capital—respondents participate in fewer mainstream cultural activities—compared with their younger counterparts. Finally, we also find that increasing participation in voluntary associations is significantly related to greater diversity of Indigenous cultural capital. Respondents who participate in a greater range of voluntary associations, also participate in a greater range of Indigenous cultural activities.

Overall, our findings suggest that these socially mobile Indigenous entrepreneurs across three international sites, have diverse social and cultural capital, that they have equally diverse Indigenous and White social capital, and that their Indigenous cultural capital is significantly more diverse than their mainstream cultural capital. Further, we find evidence that aspects of increasing social mobility are positively and significantly related to increasingly diverse Indigenous and mainstream social capital, and to increasingly diverse Indigenous cultural capital.

We also note that there are some differences in the diversity of social and cultural capital related to research site, suggesting that there are characteristics unique to Toronto, Phoenix, and Brisbane that may create opportunities to participate in a more or less diverse range of cultural activities, and to know people of different backgrounds to varying degrees.

Although this current article is not about Indigenous definitions of business success, it is clear through interviews with these entrepreneurs that their Indigenous social and cultural capital is incredibly important to them, and considered to be a key feature of their business success. They also see their business success as a mechanism to participate in and give back to their communities. In this respect, Indigenous entrepreneurs are very clear about the importance of maintaining their Indigenous social and cultural capital, even as they become more socially mobile and their businesses do well.

## Discussion

Census data from the United States, Australia, and Canada clearly show that the social mobility of Indigenous people is an empirical phenomenon—there are a greater number of people living in cities, graduating with university degrees, earning above median incomes, working full-time, starting businesses, and owning their own homes.

The core concern of this article is to look at the relationship between indicators of social mobility, and social and cultural capital. We find support that runs contrary to current theory that argues as Indigenous people gain in levels of income and occupational prestige, there is a decrease in their connections to Indigenous social networks and culture. We find evidence for the opposite, fitting with generalized arguments around social and cultural capital, where individuals who work in higher prestige occupations, and who have higher incomes, have more diverse social networks (Angelusz & Tardos, 2001; Erickson, 2004). They are not only more sought after as sources of social capital themselves, but are also able to open more doors on their own.

Using evidence from qualitative interviews, our findings are further bolstered by entrepreneurs’ statements about the importance of Indigenous social and cultural capital as a key aspect of business success. The research presented in this article shows that Indigenous entrepreneurs across the three research sites have a more holistic understanding of success (and wealth) that prioritizes strong and active connections to Indigenous networks and culture. Rather than foregoing their Indigenous networks and cultural practices as they become increasingly socially mobile and successful as business owners, respondents are using their status to maintain (if not grow) their connections.

When it comes to effects of business attributes, we find a significant and positive relationship between doing business nationally and internationally, Indigenous social capital, and Indigenous and mainstream cultural capital. This is likely less to do with where entrepreneurs are doing business, and more about the kinds of companies they run. Large, national and international corporations tend to employ many more people, have greater profit, and in turn, greater incomes and prestige associated with them. Indigenous entrepreneurs, as noted in qualitative interviews, also try to prioritize jobs to community members, friends and family. Both lend weight to explaining the effect of market reach on not only increasing the diversity of Indigenous social capital, but the ability to participate in a greater range of cultural activities.

That a greater use of formal business assistance programs is associated with a decline in the diversity of White social capital, suggests there is something about these programs that creates opportunities that perhaps rely less on ties to Whites in these urban economies. Without a similar increase in diversity of ties to Indigenous people or non-Whites/non-Indigenous ties, it is difficult to suggest a possible explanation. Further research about this relationship is needed.

There are also differences across research sites. This is not surprising—there are differences in the composition of populations, policies, legal status, and proximity of Indigenous communities that likely contribute to the differences we are findings. Of significant importance, is the relative proximity of Indigenous communities to Toronto and Phoenix, created through policies meant to displace and colonize Indigenous Peoples in North America through reserve/reservation systems.

Within 1 to 2 hr drive of Toronto, there are several First Nations that have created opportunities and investment on reserve, and now have significant levels of economic development happening. Similarly, with Phoenix, there are two Native American reservation communities that butt up against city limits, and several others within 1 to 2 hr drive of the city, all with varying degrees of economic development. We suggest that these have served as mechanisms to facilitate the maintenance and development of Indigenous social capital for respondents living in these two cities, compared with entrepreneurs in Brisbane, where similar bounded Indigenous communities were not created as a product of colonization, nor are necessarily as close to the city.

Some respondent attributes also see an effect on social and cultural capital. Interestingly, respondents who have marital or common-law partners also have more diverse ties to individuals of European ancestry. Higher rates of inter-marriage are the likely reason for this finding. We also see a negative effect of age on participation in a greater range of mainstream cultural activities. It is possible that loss of mobility, declining interest and opportunity may be responsible for this effect. We also find that increased participation in voluntary associations is linked to increased participation in different kinds of Indigenous cultural activities. Many voluntary associations relate to arts and culture, encouraging and facilitating participation. It is likely the case here.

What is perhaps surprising, is the lack of diverse connections to new immigrant groups across all three research sites, as well as a similar lack of participation across other ethnic cultural activities. Whether this is a product of lack of opportunity or interest, or simply a greater focus on developing and maintaining ties to social networks and culture in other arenas, further work needs to be encouraged, to get at the reasons behind Indigenous entrepreneurs’ lack of engagement.

Throughout this article, we have chosen to remain consistent with existing literature, and use contemporary understandings of social mobility and forms of capital to provide a much-needed contribution to this space. It is also imperative that we call for further engagement between scholarly knowledge production and lived Indigenous experience of social mobility to decolonize our greater understanding of what these concepts mean to Indigenous Peoples. This demands greater engagement with socially mobile Indigenous individuals who are working to assert their worldview and values toward building symbolic power and institutional legitimacy through and for Indigenous social and cultural capital.

Of note, consumption and reproduction of cultural capital is not a purely assimilatory process for Indigenous people, but one that is open to resistance, reframing, and negotiation (Ashcroft, 2013; Xu, 2018) explores the conversion of colonized cultural capital into anti-colonial cultural capital by Indigenous actors. Mastering tools for the communication and negotiation of social space for Indigenous people is at the heart of the sophistication of Indigenous cultural capital (Xu, 2018, p. 10). For Indigenous entrepreneurs, turning one’s mainstream social and cultural capital resources toward business needs can enable access to capital investment, customers, and clients (Alexy et al., 2012).

Although a perhaps misplaced skepticism exists for those Indigenous people who have become socially mobile, especially entrepreneurs (Behrendt, 2015; Bunten, 2011; Wahlquist, 2016), and their ability to stay connected, we cannot afford to be ahistorical about the challenging work required to build wealth, and on the consequences to individual and collective perceptions of success. In this respect, what is far less understood is the nature of Indigenous cultural capital in the support of Indigenous entrepreneurial advancement and social mobility.

To this end, the current work contributes to conversations around our understanding of entrepreneurship, social mobility, and social and cultural capital. We take up the call for an expanded understanding of Indigenous entrepreneurship by examining the distinctiveness of social and cultural capital through the lens of Indigenous entrepreneurs (Peredo & McLean, 2013). Furthermore, in response to Bapuji et al. (2020), we bring forward this article in an under-researched topic, Indigenous entrepreneurial experiences of social and cultural capital.

Importantly, this empirical phenomenon of Indigenous social mobility is set against statistics that report Indigenous households experiencing persistent income poverty (Walter & Saggers, 2020). While we are seeing positive statistical and data representations of Indigenous social mobility, the lived experience of Indigenous people in Canada, the United States, and Australia continues to be hampered by unequal settler/Indigenous power relations (Langton, 2012; Walter, 2009), generating a politically catalyzed dichotomy between socially mobile Indigenous people and Indigenous people experiencing persistent poverty. The lived reality of Indigenous entrepreneurs, as they create value and find ways to augment meaning of trading in contemporary capitalism, produces a more holistic and complicated understanding of Indigenous experiences of social and cultural capital (Bapuji et al., 2020; Peredo & McLean, 2013).

Dissociative effects of the lived reality of Indigenous social mobility require a new research agenda, that explores not just the objective markers of mobility but the social, cultural and emotional dimensions of the lived experience of mobility (Friedman, 2014, p. 364). Understanding the psychological price of social mobility for Indigenous people and the contradictory implications for cultural identity (Friedman, 2016) requires sophisticated analysis that brings Indigenous knowledges into dialogue with the colonial and “paradoxical social structure” of individualization (Beck, 2001, p. 277).

Ideally, this article calls for a move away from “damage-centered” research questions (Tuck, 2009) that perpetuate colonial definitions of who Indigenous people are. Future research efforts need to continue exploring questions of how Indigenous peoples are leveraging their social mobility and the interaction of the relationship between these experiences and connections to Indigenous social networks and culture. The causal nature of the relationships explored with this article is complex and would benefit greatly from committed longitudinal work with a diverse sample of Indigenous people. There is also a need to focus on the strengths of community cultural wealth of Indigenous people, communities, and networks and what this brings to broader understandings of social mobility (Yosso, 2005). Considering Indigenous experiences requires us to look deeper at how the theory of social mobility operates, the role social structure plays in perpetuating and maintaining hegemonic understandings of Indigenous Peoples, their relationship to social mobility, and the de-legitimization of Indigenous forms of capital.

## Conclusion

This article starts with three clear questions: (a) are Indigenous entrepreneurs, as members of a socially mobile class, cultivating rich and varied social and cultural capital; (b) do mainstream forms of capital come at the expense of Indigenous forms of capital; (c) how is social mobility related to social and cultural capital? Using data collected from a three-country study of Indigenous entrepreneurs, our central findings show that respondents have equally diverse Indigenous and White/mainstream social capital, and that they also have significantly more diverse Indigenous cultural capital, when compared with mainstream cultural capital.

Second, entrepreneurs prioritize their connections to their Indigenous communities, families and friends as a measure of success, demonstrating that Indigenous forms of capital are not being sacrificed with participation in mainstream contexts. Finally, increases in income and occupational prestige are positively and significantly related to diverse Indigenous social and cultural capital, as well as diverse ties to Whites. In this respect, indicators of social mobility are positively associated with some forms of social and cultural capital, lending support (and running contrary) to the idea that being socially mobile is not eroding Indigenous people’s connections to family, friends, and culture. Further work is required.
